# Factors Influencing the Preventive Practice of International Students in South Korea against COVID-19 during the Pandemic

**DOI:** 10.3390/ijerph18052259

**Published:** 2021-02-25

**Authors:** Gun Ja Jang, Ginam Jang, Sangjin Ko

**Affiliations:** 1 Department of Nursing, Daegu University, Daegu 42400, Korea; kjjang14@daegu.ac.kr; 2 School of International Studies, Yeungnam University, Gyeongsan 38541, Korea; jjggnn@hanmail.net; 3 Department of Nursing, University of Ulsan, Ulsan 44610, Korea

**Keywords:** coronavirus, infection control, quarantine, students

## Abstract

As the novel coronavirus disease (COVID-19) spreads worldwide, quarantine guidelines are being constantly updating to prevent the transmission of this virus. Regardless of which country international students live in, they might receive limited crucial quarantine guidelines from that country’s government. The purpose of this study was to identify factors influencing the preventive practice of international students in South Korea during the COVID-19 pandemic. This was a cross-sectional descriptive study. Data were collected from international students in three universities from July 10 to July 31 in 2020. A total of 261 international students participated in the survey, using an online questionnaire. Data were analyzed by independent t-test, one-way ANOVA, Pearson correlation coefficients, and multiple regression analysis. Preventive practice during the COVID-19 pandemic was affected by duration of stay in Korea (β = −0.21, *p* < 0.001), attitudes (β = 0.22, *p* = 0.001), and trust in Korea’s quarantine system (β = 0.33, *p* < 0.001). This study showed that attitudes and trust in the quarantine system could affect personal preventive practice during the outbreak of a highly contagious disease such as COVID-19.

## 1. Introduction

The novel coronavirus disease 2019 (COVID-19), also called severe acute respiratory syndrome coronavirus 2 (SARS-CoV-2), is the cause of an ongoing worldwide pandemic [1]. Its main symptoms include fever, cough, sore throat, and many other respiratory symptoms. It was first reported in those with pneumonia of unknown cause in Wuhan, China, in December 2019 [1]. In Korea, the first COVID-19 patient was a 35-year-old Chinese woman who arrived from Wuhan on 19 January 2020 [2]. Despite the imposition of Wuhan’s entry restrictions on 4 February, the number of confirmed COVID-19 cases in Korea sharply increased, starting with the 31st identified patient in Daegu city, a city with 2.5 million residents [3]. The number of patients reached 7755 in Korea on 12 March 2020, with the country ranking second globally. As COVID-19 can spread very rapidly in a specific cluster (Christchurch), 87.3% of all confirmed South Korean cases occurred only in Daegu and the adjacent Gyeongbuk province, as of 17 March 2020 [3,4].

It is essential to know and adhere to proper prevention guidelines for infection control. During the emergence of an unknown novel virus, the guidelines from the World Health Organization (WHO) [5], Centers for Disease Control and Prevention (CDC) [6], and Korea Disease Control and Prevention Agency (KDCA) [7] continue to be updated and revised. However, sometimes, the information provided in these guidelines is confusing or conflicting [8]. Therefore, it is difficult for most international students living in Daegu and Gyeongbuk to keep track of the continually changing guidelines announced by Korea’s government or to find relevant information. Mostly, many international students often obtain COVID-19 related information through social media [9], so they need to be managed to avoid exposure to misinformation, particularly during the COVID-19 pandemic. Most of them are non-English speakers and may have difficulty understanding the official announcements provided in Korean and English. For a highly contagious disease such as COVID-19, individuals’ prevention practices are of the utmost importance in preventing infectious diseases, and it is necessary to investigate the preventive practices of international students, who are often overlooked, during quarantine.

According to the Knowledge–Attitude–Practice (KAP) model, knowledge allows individuals to change their attitudes, and finally, to change their practices [10]. Each person’s compliance with preventive guidelines is mostly affected by their KAP toward COVID-19 under KAP theory [11]. In previous studies about infectious diseases, including Middle East respiratory syndrome (MERS) [12] and severe acute respiratory syndrome (SARS) [13], the knowledge and attitudes were found to be correlating variables with preventive practice. A few KAP studies on COVID-19 reported similar findings, and most of them were conducted on native college or university students majoring in nursing or medicine [14,15,16], which may be not be generalizable to international students. Moreover, very few studies have explored the relationship between trust in a quarantine system and preventive practice. Therefore, the aim of this study was, for international students who are likely to be marginalized by the national quarantine system, to investigate the relationship between KAP towards COVID-19 and influencing factors of preventive practice.

## 2. Methods

### 2.1. Study Design

This study utilized a cross-sectional descriptive study design. It was conducted in two steps. First, a pilot study was conducted to test the feasibility and reliability of the measuring instruments. In particular, whether international students could entirely understand a questionnaire written in Korean was assessed. After completing the pilot study, the primary survey was conducted to identify factors influencing international students’ preventive practice during the COVID-19 pandemic. The questionnaire developed by researchers consisted of demographic characteristics, knowledge, attitudes, trust in Korea’s quarantine system, and preventive practice related to the COVID-19 pandemic.

### 2.2. Participants

Participants were conveniently sampled, and they were voluntarily recruited through a recruitment announcement from three (Y, D, another D) universities located in Daegu and Gyeongbuk, Korea. These universities are private universities with 68, 91, and 37 departments, respectively, and Korean language education centers. Around 2900 international students are registered in the three universities, which are the leading universities in terms of the number of international students enrolled [17]. The number of eligible participants was 1200 based on the inclusion and exclusion criteria. Among them, 300 international students participated in the online survey, and 43 were excluded due to unresponsive items. Finally, data from 256 international students were used for statistical analysis. Inclusion criteria were as follows: (1) those who registered in a university or a language school of a university and took online or offline lectures; (2) those who had a level 3 in the Test of Proficiency in Korean (TOPIK) [18] that allowed international students to be accepted by a Korean university for studying as a minimal requirement; (3) those who had been living in South Korea for more than six months and voluntarily participated in the online survey. Exclusion criteria were as follows: (1) age of younger than 20 years or more than 30 years; and (2) those who had a regular job as foreign workers, although they were studying in a university.

### 2.3. Measures

#### 2.3.1. Knowledge about COVID-19 Pandemic

The researchers developed a COVID-19 pandemic knowledge scale based on the CDC’s guidelines [6] and the questions used to survey MERS-related knowledge among Korean nursing students [19]. This scale consisted of 10 items. Its content validity index (CVI) was rated by three nursing professors using a 4-point Likert scale (1 = not relevant, 4 = very relevant). In the pilot study, ten foreign students were asked to respond to some words in vague terms (“droplet infection” and “olfactory paralysis”). Subcategories were onset (1 item), symptoms (4 items), transmission (3 items), and prevention (2 items). A correct answer was given 1 point, and an incorrect answer or “do not know” response was given 0 points, with a higher score indicating a higher level of knowledge. The final CVI of the scale was 0.88. Its reliability (Kuder–Richardson 20) was 0.73.

#### 2.3.2. Attitudes toward the COVID-19 Pandemic

The researchers developed five items relating to attitudes toward the COVID-19 pandemic by referring to previous studies [20,21]. Each item was rated on a 5-point Likert scale from “Not at all” (1 point) to “Absolutely yes” (5 points), with a higher score indicating that the participant was more likely to feel that that the COVID-19 pandemic was a severe health issue. Ambiguous words and phrases were revised through a pilot study. Cronbach’s α of the scale in the primary study was 0.72.

#### 2.3.3. Trust in Korea’s Quarantine System during the COVID-19 Pandemic

The researchers developed three items relating to trust in Korea’s quarantine system during the COVID-19 pandemic. Each item was rated on a 5-point Likert scale (1 = “Not at all” to 5 = “Absolutely yes”). Regarding the reliability of the scale, Cronbach’s α in the primary study was 0.86.

#### 2.3.4. Preventive Practice during the COVID-19 Pandemic

Preventive behaviors during the COVID-19 pandemic are related to the respondents’ practice behaviors to prevent the transmission of the disease during the previous two weeks. The scale, based on CDC guidelines [6] and developed by the researchers, consisted of ten items. Three nursing professors tested the scale, and its CVI was 0.89. Each item could be rated on a 4-point Likert scale (0 = “Not performed” or ”Not applicable” to 3 = “Performed all the time”), with a higher score meaning greater performance of the preventive practice. The reliability (Cronbach’s α) of the scale was 0.86 in the primary study.

### 2.4. Data Collection

Before developing scales for this study, the researchers completed an online course entitled COVID-19 Contact Tracing, authorized by Johns Hopkins University, to guarantee researchers’ expertise regarding COVID-19. This study was approved by the Institutional Review Board (IRB) of U University in Korea (No. 1040968-A-2020-009).

A pilot study was conducted from 3 July to 7 July 2020, to correct these scales. The primary study was conducted from 10 July to 31 July 2020. International students were notified and encouraged to participate in the study through a voice recording or an announcement via the three universities’ Learning Management Systems. If they intended to participate in the study, they could follow a link to access the survey. The first page of the online survey included the consent to participate in the research, and all subjects proceeded to the survey after agreeing to participate in the research.

### 2.5. Data Analysis

Data of 256 participants were analyzed using SPSS/WIN 25.0 software. General characteristics and knowledge about COVID-19 were expressed as mean, standard deviation, frequencies, and percentages. Differences in preventive practice according to general characteristics were analyzed using t-test, ANOVA, and post-hoc Scheffe test. Attitudes, trust in Korea’s quarantine system, and preventive practice during COVID-19 were presented as mean, standard deviation, and minimum–maximum values. Correlation between study variables was analyzed using Pearson’s correlation test. Factors influencing COVID-19 preventive practice were identified by hierarchical multiple linear regression analysis.

## 3. Results

### 3.1. Preventive Practice According to General Characteristics

Participants’ average age was 22.90 years, and there were slightly more males (50.8%) than females (49.2%). There were more undergraduate students (56.3%) than language school students (43.8%) in the affiliation. Regarding their duration of stay in Korea, durations of 1 to 2 years were the most common (49.6%), and a duration of more than three years (9.8%) represented the lowest proportion. As for nationality, Vietnamese (43.0%) represented a higher proportion than Uzbekistani (27.0%), Chinese (18.8%), and other nationalities (11.3%). Other countries included Japan, the Philippines, and Malaysia.

There were significant differences in preventive practice by gender (t = 4.19, *p* < 0.001), affiliation (t = 5.47, *p* < 0.001), and duration of stay in Korea (F = 8.93, *p* < 0.001). As a result of the post-hoc comparison test, students who stayed for 1 to 2 years practiced significantly lower preventive behaviors than those who stayed for more than two years (Table 1).

### 3.2. Knowledge about COVID-19

The total correct rate was 85.5% for knowledge about COVID-19 among international students in South Korea. The item with the highest correct answer rate (99.2%) was about prevalent symptoms such as fever, cough, and a sore throat in the symptoms category. However, students most lacked information on the two-week quarantine period in the transmission category (57.0%) (Table 2).

### 3.3. Attitudes, Trust in Korea’s Quarantine System, and Preventive Practice for COVID-19

The mean score of participants’ attitudes toward COVID-19 was 4.40 out of 5. The highest scoring item (4.41) was about social distancing, and the lowest scoring item (3.04) was about recognition of the dangers of COVID-19.

The mean score of participants’ trust in Korea’s quarantine system was 4.13 out of 5. They were satisfied with the order of the healthcare system (score of 4.26), government quarantine system (score of 4.10), and local government quarantine system (score of 4.01).

The mean score of their preventive practice was 2.54 out of 3. The highest score was 2.82 for the item about wearing a face mask in public, and the lowest score was 2.29 for the item of avoiding touching one’s own face with unwashed hands (Table 3).

### 3.4. Correlations among Variables

Knowledge, attitude, and trust in Korea’s quarantine system were positively correlated with each other. Preventive practice for COVID-19 showed significantly positive correlations with knowledge (r = 0.29, *p* < 0.001), attitudes (r = 0.46, *p* < 0.001), and trust in Korea’s quarantine system (r = 0.48, *p* < 0.001) (Table 4).

### 3.5. Factors Influencing Preventive Practice against COVID-19

A hierarchical multiple linear regression was conducted to analyze factors influencing preventive practice. The Durbin-Watson statistic showed an approximate value of 2, indicating no problem assuming the independence of residuals. The variance expansion index was all less than 10, indicating that there was no multicollinearity problem. In model 1, regression analysis was performed by gender, affiliation, and Stay duration in Korea, significant variables among general characteristics, as dummy variables. Regression equation was significant (F = 22.15, *p* < 0.001) and the explained power was 14%. Gender (β = 0.19, *p* = 0.001) and stay duration in Korea (β = −0.30, *p* < 0.001) were significant variables. In model 2, preventive practice against COVID-19 was affected by the stay duration in Korea (β = −0.21, *p* < 0.001), attitudes (β = 0.22, *p* = 0.001), and trust in Korea’s quarantine system (β = 0.33, *p* < 0.001). Final regression equation was significant (F = 26.58, *p* < 0.001) and the explained power was 33% (Table 5).

## 4. Discussion

The highly infectious nature of COVID-19 is causing dramatic changes in the lifestyles of millions of people worldwide. In particular, in South Korea, the spread of COVID-19 resulted in a dramatic increase in the number of confirmed cases per day, reaching 741 in Daegu only ten days after discovering the 31st patient in Daegu on 18 February 2020 [3,4]. At that time, Daegu and Gyeongbuk had to fight a novel epidemic without being prepared. Above all, international students, mostly from non-English-speaking countries, had difficulty obtaining information from a daily updated national policy for novel infectious diseases. Therefore, this study was performed to identify influencing factors associated with preventive practice during the COVID-19 pandemic for international students who might have been marginalized during information transmission. There have been very few studies on preventive practice during COVID-19 among international students, to our knowledge.

According to the KAP model, knowledge, attitudes, and practice are inter-related [9,10], and previous studies have found that knowledge is the most crucial factor in preventive behavior regarding novel influenza A [22]. However, the result of the final hierarchical regression analysis in this study found that participants’ attitudes, but not their knowledge about COVID-19, affected their preventative practice, although they also showed significant positive relationships [23,24,25].

Knowledge about COVID-19 was moderate (85.5%), and four out of 10 items showed a correct answer rate of less than 80%. This study’s knowledge level was lower than that of 300 international students in Hubei province in China, which showed only two out of 13 items were correctly answered, at a rate of less than 80% [11]. It also was lower than that of 93.2%, measured in 474 nursing students in the study of Sun et al. [26], and 90%, measured in 6910 residents in China [20]. Of course, a direct comparison might be difficult due to differences in the instruments used and the different populations.

These results indicate that it is difficult for international students to obtain accurate, newly updated information and national policy. In particular, students lacked information on the two-week quarantine period (57.0%) and the absence of a COVID-19 vaccine (as of June 2020) (62.1%). However, results showed a very high correct answer rate in regard to wearing a face mask to prevent COVID-19 (98.4%), and in regard to preventive practice during the previous two weeks, wearing a face mask in public achieved the highest score. This result was higher than that of 58.2%, measured in the Iranian population, who responded that they always wear a mask [27]. Because COVID-19 is transmitted via droplets [6], personal preventive equipment (PPE), especially face masks, can play a critical role in controlling COVID-19 [28]. This is thought to be because KDCA actively encouraged and promoted the wearing of face masks [7]. Regarding the attitudes, subjects agreed that disease prevention would be possible if they complied with quarantine regulations, including social distancing. Moreover, their trust in Korea’s quarantine system was high (score of 4.13). In March, the Korean government attempted to prevent the spread of COVID-19 by introducing a 5-day rotation system for mask distribution so that people can purchase disposable face masks each week, by sending text messages about safety guidance, such as wearing a mask, social distancing, and hand washing, and by briefing residents on the situation twice a day on television [29]. It was believed that such an active response from the government improved international students’ trust in the quarantine system. Direct comparison is difficult because there is no previous study on the correlation between preventive practice and the quarantine system. However, people with low trust in the healthcare system are associated with low adherence to human immunodeficiency virus (HIV) care and poorer health outcomes [30]. Therefore, having trust in quarantine systems and demonstrating compliance with guidelines might be more important than having a high level of knowledge regarding factors affecting preventative practice.

## 5. Conclusions

In this study, international students residing in Daegu and Gyeongbuk, with the highest number of confirmed COVID-19 cases in Korea, were investigated for factors affecting preventive practice regarding COVID-19. Results indicate that having the right attitudes and trust in the public quarantine system are more important in maintaining individual preventive actions than having the right knowledge about infectious diseases in a pandemic situation.

This study has some limitations. First, many participants in this study were Asian and from the southeast or northeast regions, so the findings were limited and cannot be generalized to all international students from all continents. However, to ensure the samples’ representativeness, we sought to secure the number of samples according to international students’ national distribution. Second, participants in this study were limited to international students enrolled in three universities, so international students who left Korea before the COVID-19 pandemic and were in their home countries were omitted. This was because they were international students who did not experience Korea’s quarantine system and prevention guidelines in the context of the COVID-19 pandemic and did not match this study’s purpose. Despite some limitations, this study was meaningful in that it sought ways to promote the preventive practices of international students living in regions where the number of confirmed cases was increasing and the fear of residents was high.

## Figures and Tables

**Table 1:** Preventive practice according to general characteristics (*N* = 256).

Variables	Categories	Mean ± SD or n (%)	Preventive Practice		
			Mean ± SD	t or F	*p* (post-hoc)
Age (yrs)		22.90 ± 2.41			
Gender	Male	130 (50.8)	2.66 ± 0.44	4.19	<0.001
	Female	126 (49.2)	2.42 ± 0.46		
Affiliation	Undergraduate	144 (56.3)	2.68 ± 0.37	5.47	<0.001
	Language school	112 (43.8)	2.36 ± 0.51		
Stay duration in Korea	Less than 1	56 (21.9)	2.58 ± 0.43	8.93	<0.001
(yrs)	1-2 ^a^	127 (49.6)	2.41 ± 0.49		(a < b,c)
	2-3 ^b^	48 (18.8)	2.71 ± 0.38		
	More than 3 ^c^	25 (9.8)	2.79 ± 0.30		
Nationality	Vietnam	110 (43.0)			
	Uzbekistan	69 (27.0)			
	China	48 (18.8)			
	Others	29 (11.3)			

**Table 2:** Knowledge about COVID-19 (*N* = 256).

Items	Answer	Category	Correct
			n (%)
1. COVID-19 is a respiratory infectious disease caused by coronavirus (SARS-CoV-2)	True	Onset	247 (96.5)
2. COVID-19 is transmitted by close contact with the infected person	True	Symptoms	249 (97.3)
3. Fever, cough, a sore throat, muscle pains, and shortness of breath are possible symptoms of COVID-19	True	Symptoms	254 (99.2)
4. Nausea, diarrhea, taste, and olfactory dysfunction are possible non-specific symptoms of COVID-19	True	Symptoms	203 (79.3)
5. It requires people who have recently had close contact with someone with COVID-19 to go into quarantine for 1 week	False	Transmission	146 (57.0)
6. COVID-19 vaccine is available in markets	False^1^	Prevention	159 (62.1)
7. Using face masks can help in the prevention of disease transmission	True	Transmission	252 (98.4)
8. COVID-19 could be fatal only for the elderly	False	Transmission	201 (78.5)
9. You could have been infected and spread COVID-19 to others even if you do not have symptoms	True	Symptoms	232 (90.6)
10. If soap and water are not readily available, use a hand sanitizer that contains at least 60% of alcohol	True	Prevention	237 (92.6)
Total			85.5%

^1^ It was scored false because there was no vaccine during the data collection period in June 2020.

**Table 3:** Attitudes, trust in Korea’s quarantine system, and preventive practice related to COVID-19 (*N* = 256).

Variables	Mean ± SD	Min-Max
**Attitudes**	4.40 ± 0.57	3–5
1. Do you have confidence that we can win the battle against the COVID-19 virus?	4.31 ± 0.97	1–5
2. Do you have confidence that you can prevent COVID-19 infection by following the precautions well?	4.39 ± 0.75	2–5
3. Do you carefully read and follow the instructions on COVID-19 from the (provincial) government and university?	4.40 ± 0.83	1–5
4. COVID-19 is a very dangerous contagious disease	3.04 ± 1.49	1–5
5. The activities in my daily life needs to be limited to prevent COVID-19	4.41 ± 0.79	2–5
**Trust in Korea’s quarantine system**	4.13 ± 0.81	2–5
1. In general, are you satisfied with how the government handles the COVID-19 outbreak?	4.10 ± 0.95	1–5
2. In general, are you satisfied with how your provincial government handles the COVID-19 outbreak?	4.04 ± 0.97	1–5
3. In general, are you satisfied with healthcare system in South Korea?	4.26 ± 0.82	2–5
**Preventive practice (During past 2 weeks)**	2.54 ± 0.47	1–3
1. Have washed my hands often with soap and water for at least 20 seconds	2.49 ± 0.70	0–3
2. Have avoided touching my eyes, nose, and mouth with unwashed hands	2.29 ± 0.89	0–3
3. Have avoided close contact with people who are sick, even inside my home	2.41 ± 0.84	0–3
4. Have stayed out of crowded places and avoided mass gatherings	2.50 ± 0.73	0–3
5. Have worn a face mask when I have to go out in public	2.82 ± 0.44	1–3
6. Have covered my mouth and nose with a tissue when I coughed or sneezed or used the inside of my elbow, if I did not have a mask on in a private setting	2.59 ± 0.70	0–3
7. Have thrown used tissues in the trash, and immediately washed my hands after coughing or sneezing	2.60 ± 0.64	1–3
8. Have cleaned and disinfected frequently touched surfaces on daily basis	2.42 ± 0.74	0–3
9. Have been alert for related symptoms	2.69 ± 0.53	0–3
10. Have taken temperature if symptoms were shown	2.61 ± 0.65	0–3

**Table 4:** Correlations among variables (*N* = 256).

Variables	Attitudes	Trust in Korea’s Quarantine System	Preventive Practice
Knowledge	0.44 (<0.001)	0.36 (<0.001)	0.29 (<0.001)
Attitudes		0.46 (<0.001)	0.46 (<0.001)
Trust in Korea’s quarantine system			0.48 (<0.001)

**Table 5:** Factors influencing preventive practice against COVID-19 (*N* = 256).

Variables	B	SE	beta	t	*p*
Model 1					
Constant	2.57	0.05		52.79	<0.001
Gender (Ref. male)	0.18	0.06	0.19	3.28	0.001
Stay duration in Korea (Ref. 1 to 2 years)	−0.28	0.06	−0.30	−5.01	<0.001
	R^2^ = 0.15, adj.R^2^ = 0.14, F = 22.15, *p* < 0.001				
Model 2					
Constant	0.94	0.23		4.14	<0.001
Gender (Ref. male)	0.03	0.05	0.03	0.54	0.593
Stay duration in Korea (Ref. 1 to 2 years)	−0.20	0.05	−0.21	−3.94	<0.001
Knowledge	0.12	0.22	0.03	0.56	0.579
Attitudes	0.18	0.05	0.22	3.49	0.001
Trust in Korea’s quarantine system	0.19	0.04	0.33	5.46	<0.001
	R^2^ = 0.35, adj.R^2^ = 0.33, F = 26.58, *p* < 0.001				
